# On accumulated degree day and maggot age calculations in Rivers and Dahlem (2023) The Science of Forensic Entomology, 2nd edition

**DOI:** 10.1093/jme/tjae065

**Published:** 2024-05-09

**Authors:** Jeffrey D Wells

**Affiliations:** Department of Biological Sciences, Florida International University, 11200 SW 8^th^ ST, Miami, FL 33199, USA

“To meet the core assumption of degree day models, a regression of degree day accumulations [for a particular amount of development at each of a range of temperatures] must be linear and have no slope” (Roe and Higley 2015).

A common task for a forensic entomologist is to shed light on the postmortem interval by estimating the age of an immature insect (e.g., a blow fly maggot) from a corpse (Greenberg 1991). This is usually done by comparison to insects of known age from a laboratory growth experiment (Wells and LaMotte 2020). Because insect development rate is profoundly affected by temperature, and because the reference data were almost never produced using the temperature history of the corpse, forensic entomologists often adopt the thermal summation concept long used in other scientific fields (see reviews by Wagner et al. 1984, Higley and Haskell 2001). If the relationship between temperature and development rate is both positive and linear, usually approximately true if it is not too hot or cold, a given amount of development (e.g., from oviposition to the second larval molt) will require the same number of accumulated degree days (ADDs) or accumulated degree hours (ADHs) independent of the insect’s temperature history. In graphical terms, ADD/ADH is the area under the plot of temperature history above a threshold (Higley and Haskell 2001).

While the forensic entomological literature includes comprehensive reviews of ADD theory (Higley and Haskell 2001, Roe and Higley 2015, Tarone and Benoit 2020), typical discussions of how to perform ADD calculations in casework lack detail (e.g., Greenberg 1985, Huntington and Higley 2008, Gennard 2012, but see Wells 2023). For that reason, and because of concerns expressed in the conclusion of this letter, I wish to correct errors in a recent publication describing forensic entomology ADD calculation methods in detail (Rivers and Dahlem 2023).

In section 14.4.3.1 of their textbook, Rivers and Dahlem (2023) (R&D) illustrated the use of ADD to extrapolate from observed development at one temperature to predicted development rate at another temperature, but instead of assuming a constant ADD value to calculate a new development time, they assumed a constant development time to calculate a new ADD. More specifically, to predict *Protophormia terranovae* development at 25 °C (for which development rate data are unavailable), they used laboratory data for this species at 23 °C and a threshold of 12. 5°C (Greenberg and Tantawi 1993). The authors assumed that the egg stage at 25 °C lasts the same number of hours as at 23 °C (16.8) and then used that value to calculate a new ADD value for the egg stage at 25 °C.

ADD [at 25 °C] = 16.8/24 * (25 − 12.5)

= 8.75

If one were to use the conventional strategy of assuming ADD to be the same at both temperatures, stage duration in time is solved as follows.

7.35 [observed ADD at 23 °C] = Duration of egg stage [at 25° C]/24 * (25 − 12.5)

Therefore: Duration of egg stage = 24 * 7.35/(25 − 12.5)

= 14.1 h

Thus we get the expected result that development time is less at a higher temperature.

In section 14.4.5, R&D expanded their hypothetical example to a case study. The forensic entomologist estimates the age of a feeding third larval instar *P*rotophormia *terraenovae* from a corpse by summing the durations of individual life stages as observed in the lab. Here the problem is not how ADD was used to extrapolate between temperatures. Rather it is that R&D summed the durations of the egg, first larva, second larva, and third larva. This describes the amount of time for the insect to become a pupa rather than a third larva.

These errors were discovered prior to publication. I corresponded with Drs. Rivers and Dahlem but unfortunately too late in the publishing process to correct the final manuscript. While R&D may revise these aspects of a future edition of their book, given the central role of insect age estimation in forensic entomology, the potentially serious consequences of erroneous forensic science analysis, and the potential use of these calculations in a cross-examination to cast doubt on correct expert testimony, I believe these issues must be clarified in print now.
